# Association Between Behavioral Dysexecutive Syndrome and the Health-Related Quality of Life Among Stroke Survivors

**DOI:** 10.3389/fpsyt.2020.563930

**Published:** 2020-09-08

**Authors:** Yannis Yan Liang, Lisha Wang, Ying Yang, Yangkun Chen, Vincent C. T. Mok, Gabor S. Ungvari, Winnie C. W. Chu, Jong S. Kim, Wai-Kwong Tang

**Affiliations:** ^1^ Stroke Center and Department of Neurology, The First Affiliated Hospital, Jinan University, Guangzhou, China; ^2^ Department of Psychiatry, The Chinese University of Hong Kong, Hong Kong, Hong Kong; ^3^ Department of Neurology, Dongguan People’s Hospital, Dongguan, China; ^4^ Department of Medicine and Therapeutics, The Chinese University of Hong Kong, Hong Kong, Hong Kong; ^5^ University of Notre Dame Australia/Graylands Hospital, Perth, WA, Australia; ^6^ Department of Imaging and Interventional Radiology, The Chinese University of Hong Kong, Hong Kong, Hong Kong; ^7^ Department of Neurology, Asan Medical Center, University of Ulsan College of Medicine, Seoul, South Korea; ^8^ Shenzhen Research Institute, The Chinese University of Hong Kong, Hong Kong, Hong Kong

**Keywords:** dysexecutive syndrome, health-related quality of life, stroke, structural equation model, depression

## Abstract

**Aim:**

Behavioral dysexecutive syndrome (BDES) is one common neuropsychiatric comorbidity after stroke. Despite evidences suggesting the adverse effect of BDES on the survivors’ outcome, little is known about the association between BDES and the health-related quality of life (HRQoL) among stroke survivors and how BDES impacts the HRQoL. This study aimed to address these questions.

**Methods:**

This study included 219 patients with acute ischemic stroke consecutively admitted to a regional hospital in Hong Kong. BDES was defined as a Chinese version of the Dysexecutive Questionnaire (DEX) score of ≥20 assessed at three months after stroke. The HRQoL was assessed with the Chinese version of the Stroke-Specific Quality of Life (SSQoL) questionnaire encompassing 12 domains. Multivariate linear regression models were employed to examine the association between BDES symptoms and the SSQoL total and domain scores. Structural equation model (SEM) was further constructed to delineate the linking pathways linking BDES and the HRQoL.

**Results:**

The study sample compromised mainly older patients with mild to moderate ischemic stroke. Compared with patients without BDES, those with BDES exhibited poorer performances regarding with the summarized SSQoL (226.2 ± 18.8 vs. 200.3 ± 29.8, p < 0.001) and almost all domains. The BDES symptoms were independently contributed to the whole HRQoL (SSQoL total score) (β = −0.20, p = 0.002), specifically to the domains in personality (β = −0.34, p < 0.001), language (β = −0.22, p = 0.01), and work/productivity (β = −0.32, p < 0.001), after adjusting demographic and clinical characteristics in linear models. The impacts of the BDES symptoms on the HRQoL were mainly explained by the indirect path mediated by depression and anxiety (path coefficient = −0.27, p < 0.05) rather than physical disability, while the resting was elucidated by the path directly linking BDES to the HRQoL (path coefficient = −0.17, p < 0.05).

**Conclusion:**

The present study preliminarily demonstrated a potential association between BDES and a lower level of the HRQoL, predominantly in domains of personality, language, and work/productivityafter acute ischemic stroke. This study also offered insights into the underlying mechanisms linking BDES and the HRQoL, implicating that integrative psychological therapies were urged to achieve better HRQoL after stroke.

## Introduction

“Dysexecutive syndrome (DES)” is a term describing a group of maladaptive cognitive and behavioral function compromising goal-directed actions, adaptive responses in nonroutine, conflicting, or complex situations and tasks (1). Godefroy O et al. defined DES into two subtypes due to the distinct manifestations: behavioral dysexecutive syndrome (BDES) and cognitive dysexcutive syndrome (CDES) (2). CDES is commonly studied as “executive dysfunction” across populations. In contrast, BDES gains less attention. The concept of “BDES” emphasizes behavioral disorders such as hyperactivity or hypoactivity, stereotyped behavior, environmental dependency, disinhibition of emotion and behavior, and anosognosia (2). BDES was frequent in patients with neurological diseases including Parkinson’s disease (PD), Alzheimer disease (AD), and traumatic brain injury (TBI) (1). Although BDES is less often studied in stroke population, a prevalence of 19%–44% makes BDES as one common neuropsychiatric sequalae among stroke survivors (3, 4).

BDES may bring multiple adverse effects to old individuals or survivors of neurologic diseases. BDES was associated with depression and disability in older adults (5), and a shorter survival in patients with amyotrophic lateral sclerosis (ALS) (6). Among stroke survivors, we recently reported that patients with BDES were more likely to have worse functional independency, more severe depressive and anxious symptoms, and worse global cognitive functions (4). Moreover, dysexecutive disorders were related to poorer social functioning in patients with schizophrenia (7).

However, the relationship between BDES and stroke survivors’ Health-related quality of life (HRQoL) has not been investigated, that is considered a more comprehensive patient-related outcome measure constituted by physical, psychological, social, and environmental components (8). Thus, the current study aimed to explore the relationship between BDES and HRQoL in patients with acute ischemic stroke at the third month after the index stroke.

## Materials and Methods

### Study Population

**Figure 1:**
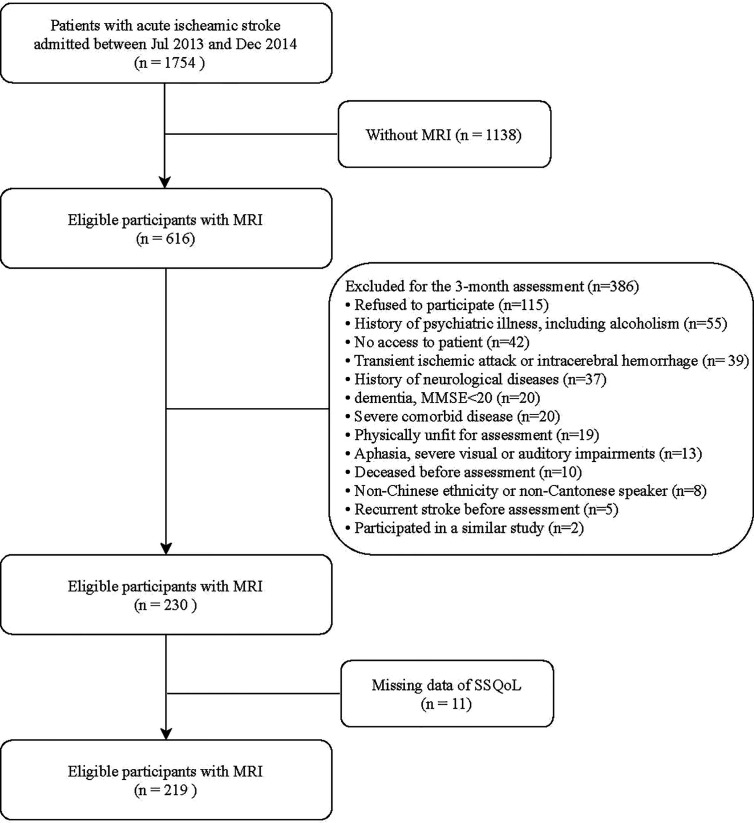
Flow chart of the enrollment.

This project belongs to the Neuropsychiatric Disorder in Stroke Registry in Hong Kong. Subjects eligible for the current study were patients with acute ischemic stroke admitted to the Stroke Unit, Prince of Wales Hospital, Hong Kong, from July 2013 to December 2014. Patients without MRI scans within 7 days of admission were excluded from the study due to the limited MRI machine time. Patients were recruited within 7 days of admission if they (1) were Chinese ethnicity; (2) adopted Cantonese as the primary language spoken; (3) aged ≥ 18 years; (4) had the ability and willingness to give consent. They were excluded if they had: (1) severe cognitive impairment, defined as an Mini-Mental State Examination (MMSE) score of <20; (2) a history of neurological diseases other than stroke and transient ischemic attack; (3) a history of psychiatric disorder such as depression, bipolar affective disorder, schizophrenia, or alcohol/substance abuse; (4) severe comorbid medical conditions, severe aphasia, auditory or visual impairments, or severe physical frailty that restricted the subjects to complete the assessments. The participants without complete data on HRQoL were further excluded from the final analysis (n = 11), therefore, leading to a total of 219 patients constituting the final sample. The flow chart of recruitment is listed in **Figure 1**.

### Ethical Statement

The study protocol was approved by the Joint Chinese University of Hong Kong-New Territories East Cluster Clinical Research Ethics Committee. All subjects signed a consent form on the day of assessment, three months after the index stroke.

### Collection of Demographic and Clinical Data

Within 7 days of the index admission, a research nurse collected demographic data (age, sex, years of education, and marital status), vascular risk factors (smoking, history of and current hypertension, hyperlipidemia, diabetes mellitus, stroke, transient ischemic attack, ischemic heart disease, and atrial fibrillation) were collected. Stroke severity was assessed with the National Institutes of Health Stroke Scale (9).

### Neuropsychiatric Assessments

At 3 months after index stroke, the following assessments were performed in a research clinic. The details of assessments are provided by our prior publications (4, 10).

Briefly, BDES was assessed with the Chinese version of the Dysexecutive Questionnaire (DEX) (11). DEX is part of the Behavioral Assessment of the Dysexecutive Syndrome (BADS) to capture the behavioral aspects of executive dysfunction (12). DEX encompasses 20 items reflecting everyday behavioral problems such as impulsivity, inhibition control, monitoring, and planning (13). DEX was appropriate to quantify BDES due to good internal consistency coefficient (0.93) and test-retest reliability (0.95), respectively (14). It has been applied to capture behavioral aspects, rather than the cognitive ones, of dysexecutive functioning in normal aging (*Relation between cognitive and behavioral aspects of dysexecutive functioning in normal aging*). In the present study, BDES was defined as an overall DEX score of ≥20 (15).

HRQoL was measured with the Chinese version of the Stroke-Specific Quality of Life (SSQoL) questionnaire. The SSQoL consists of 12 domains: energy, family roles, language, mobility, mood personality, self-care, social roles, thinking, upper-extremity function, vision, and work/productivity (16). The range of total SSQoL score is 49–245. The domain scores vary from 3 to 30.

The Hospital Anxiety Depression Scale-Anxiety Subscale (HADS-AS) and the 15-item Geriatric Depression Scale (GDS), modified Rankin Scale (mRS), Lubben Social Network Scale (LSNS), and Mini-Mental State Examination (MMSE) were adopted to evaluate anxious and depressive symptoms, physical disability, social support, and cognitive function, respectively. Of note, the GDS-15 has good sensitivity (89%) and specificity (73%) to screen depressive symptoms following stroke in Chinese elderly (17) and is able to capture less severe depressive symptoms in the elderly (18).

### MRI Data

All participants received brain MRI scans using a 1.5-Tesla scanner (Sonata; Siemens Medical) within 7 days of admission. The MRI data of following sequences were collected: diffusion-weighted images, T2 gradient echo, T1- and T2-weighted, and fluid attenuated inversion recovery sequences. A qualified neurologist (YC) who was blind to all clinical data and assessments manually calculated the volumes of acute infarcts. Briefly, the contour of an acute infarct with restricted water diffusion identified on diffusion-weighted imaging with b-values of 1,000 was outlined. The total volume was then generated by multiplying the total area by the sum of the slice thickness and gap.

### Statistical Analyses

All analyses were conducted using IBM SPSS Statistics, version 23.0 (SPSS, Inc., Chicago, IL, USA). Data are presented as means ± standard deviations (SD), medians (interquartile ranges or ranges), or proportions, as appropriate. The level of significance was set at 0.05 (two-tailed).

First, the SSQoL total score and domain scores were compared between patients with BDES and without BDES using the Chi-square test or the Fisher’s exact test, Student’s t-test, or Mann-Whitney U test, as appropriate. Second, univariate linear regressions were carried out to examine the associations between the demographic, clinical, and imaging characteristics with SSQoL total score. The variables showing a trend to be associated with the SSQoL total score (p < 0.1) (history of stroke, NIHSS on admission, volume of acute infarcts, scores of GDS, HADS-A, mRS, LSNS, and MMSE), as well as age, sex, and education, were selected as covariates into the further multivariate models. Next, a multivariate linear regression model was employed to assess the association between BDES (DEX score) and the HRQoL (total SSQoL score). All the abovementioned covariates and BDES score were forced to be entered as independent variables in the multivariate linear regression model. Similarly, the association between BDES and each domain of SSQoL was investigated. None of the pairs of covariates were found to be collinear with all r values of < 0.5 in the covariate-wise correlations.

In addition, structural equation modeling (SEM) in SPSS AMOS, Version 23.0 (IBM) was used to explore the linking pathways between BDES and the HRQoL. Binary correlations were first performed to obtain the variance-covariance matrix (default model). Next, grounded on the literature that BDES were related to depression/anxiety and disability, a fully saturated model was established to predict the HRQoL with the mediators selected from those variables significantly associated with SSQoL total score (GDS, HADSA-A, and mRS). The model parameters were subjected to a maximum likelihood estimation. Only the paths with p < 0.05 were retained to constitute the final model. Two parameters were used to judge the model fitting: (a) the comparative fit index (CFI) > 0.90 (19); (b) the root mean square error of approximation (RMSEA) <0.05 (20).

## Results

### Characteristics of the Study Sample

The final analysis included 219 participants (**Figure 1**). The demographic characteristics, stroke severity (NIHSS), and history of vascular diseases were similarly distributed between subjects included and excluded (data not shown).

The study sample mainly included the older stroke survivors with a mean age of 66.1 years old (SD = 10.8). 37.0% were women. All patients had at least one vascular risk factor. Most of them suffered from a mild to moderate ischemic stroke (NIHSS: median = 3, IQR = 1–5). The mean volume of acute infarcts was 3.6 ml (SD = 7.6). The patients received moderate level of social support (LSNS: 22.9 ± 7.7). Accordingly, most patients manifested mild to moderate level of BDES symptoms (DEX: 14.4 ± 7.7), depressive symptoms (GDS: median = 3, IQR = 1–5), anxious symptoms (HADSA-A: median = 0, IQR = 0–3), physical disability (mRS: median = 1, IQR = 1–2), and relative good cognitive function (MMSE: 26.3 ± 2.5) (**Table 1**). In this sample, the prevalence of BDES was 22.8% (50 out of 219). The SSQoL total score is 220.3 ± 24.3 (**Table 2**).

**Table 1:** Clinical and imaging characteristics of the study sample.

	Whole sample
	(n = 219)
**Demographic characteristics**	
Age, years, mean ± SD	66.1 ± 10.8
Female sex, n (%)	81 (37.0)
Education, years, median (IQR)	7.4 ± 4.1
Without spouse, n (%)	56 (25.7)
Current or prior smoker, n (%)	87 (39.7)
**Clinical characteristics on admission**	
Hypertension, n (%)	135 (62.2)
Diabetes mellitus, n (%)	57 (26.3)
Hyperlipidemia, n (%)	71 (32.7)
Previous stroke, n (%)	27 (12.4)
Transient ischemic attack, n (%)	10 (4.6)
Ischemic heart diseases, n (%)	14 (6.5)
Atrial fibrillation, n (%)	28 (12.9)
NIHSS, median (IQR)	3 (1–5)
**MRI characteristics on admission**	
Volume of acute infarcts, mean ± SD, ml	3.6 ± 7.6
**Assessments at 3-month after stroke**	
BDEX symptoms (DEX), mean ± SD	14.4 ± 7.7
Depressive symptoms (GDS), median (IQR)	3 (1-5)
Anxiety symptoms (HADS-A), median (IQR)	0 (0-3)
Physical disability (mRS), mean ± SD	1 (1-2)
Social support (LSNS), mean ± SD	22.9 ± 7.7
Cognitive function (MMSE), mean ± SD	26.3 ± 2.5

BDEX, Behavioral dysexecutive syndrome; DEX, Dysexecutive Scale; GDS, Geriatric Depression Scale; HADSA-A, Hospital Anxiety Depression Scale (Anxiety subscale); IQR, interquartile range; LSNS, Lubben Social Network Scale; MMSE, Mini-Mental State Examination; mRS, modified Rankin Scale; NIHSS, National Institutes of Health Stroke Scale; SD, standard deviation.

**Table 2:** Comparisons of the SSQoL at three months after stoke between BDEX and non-BDEX groups.

	Whole sample	BDEX	non-BDEX	P^‡^
	(n = 219)	(n = 50)	(n = 169)	
SSQoL total score, mean ± SD	220.3 ± 24.3	200.3± 29.8	226.2 ± 18.8	<0.001
***SSQoL subscores, mean ± SD***				
Energy	9.5 ± 3.8	7.7 ± 3.1	10.1 ± 3.8	<0.001
Family role	13.5 ± 2.6	11.8 ± 3.4	13.9 ± 2.1	<0.001
Language	24.5 ± 1.4	23.9 ± 2.3	24.7 ± 0.9	0.002
Mobility	27.8 ± 3.5	26.5 ± 4.5	28.1 ± 3.1	0.008
Mood	23.1 ± 4.1	19.9 ± 6	24.0 ± 2.7	<0.001
Personality	11.9 ± 3.7	9.2 ± 3.6	12.6 ± 3.4	<0.001
Self-care	24.2 ± 2.3	23.4 ± 3.2	24.5 ± 2.0	0.002
Social role	23.8 ± 3.6	21.5 ± 5.7	24.4 ± 2.3	<0.001
Upper-extremity function	12.6 ± 3.0	10.7 ± 3.4	13.2 ± 2.7	0.004
Vision	24.0 ± 2.9	23.0 ± 3.8	24.3 ± 2.5	0.063
Work	14.6 ± 1.3	14.3 ± 1.6	14.7 ± 1.2	<0.001

BDEX, Behavioral dysexecutive syndrome; IQR, interquartile range; SSQoL, Stroke-Specific Quality of Life.

^‡^Mann-Whitney U test.

### Associations Between BDES Symptoms and the HRQoL

Compared with the patients without BDES, those with BDES tended to have poorer HRQoL indicated by SSQoL total score (226.2 ± 18.8 vs. 200.3 ± 29.8, p < 0.001). Additionally, almost all aspects of SSQoL except vision were significantly worse in BDES group compared with the non-BDES group (**Table 2**). History of stroke, stroke severity (NIHSS), volume of acute infarcts, social support (LSNS), cognitive function (MMSE), depressive symptoms (GDS), anxious symptoms (HADS-A), and physical disability (mRS) were potential predictors of the HRQoL with p < 0.1 in the univariate linear regression models (**Table S1**), therefore, were regarded as covariates in the multivariate models. The multivariate linear regression models showed that the BDES symptoms (DEX total score) were significantly associated with SSQoL total score (β = −0.20, Standard Errors (SE) = 0.20, p = 0.002), and multiple domains of SSQoL including language (β = −0.22, SE = 0.02, p = 0.01), personality (β = −0.34, SE = 0.04, p < 0.001), and work/productivity (β = −0.32, SE = 0.04, p < 0.001), adjusting for age, sex, education, history of stroke, stroke severity (NIHSS), volume of acute infarcts, social support (LSNS), cognitive function (MMSE), depressive symptoms (GDS), anxious symptoms (HADS-A), and physical disability (mRS) (**Table 3**).

**Table 3:** Multivariate linear models examining the associations between BDEX and the SSQoL three months after stroke.

Predictors^b^	Model 1^a^			Model 2^a^			Model 3^a^			Model 4^a^		
	SSQoL Total			SSQoL Language			SSQoL Personality			SSQoL Work/productivity		
	β	SE	p	β	SE	p	β	SE	p	β	SE	p
BDEX symptoms	−0.20	0.20	0.002	−0.22	0.02	0.01	−0.34	0.04	<0.001	−0.32	0.04	<0.001
Depressive symptoms	−0.39	0.53	<0.001	–	–	–	–	–	–	–	–	–
Anxious symptoms	−0.14	0.48	0.02	−0.17	0.04	0.05	−0.21	0.09	0.01	–	–	–
Physical disability	−0.40	1.71	<0.001	−0.31	0.14	<0.001	–	–	–	−0.25	−0.35	<0.001
Social support	−0.14	0.17	0.01	−0.09	0.01	0.04	−0.28	0.03	<0.001	–	–	–

BDEX, Behavioral dysexecutive syndrome; SE, Standard Errors; SSQoL, Stroke-Specific Quality of Life.

a Model 1, 2, 3, and 4 were multivariate linear regressions models adjusted for age, sex, education, history of stroke, stroke severity (NIHSS on admission), volume of acute infarcts, social support (LSNS), and cognitive function (MMSE), with enter selection method.

b The significant predictors were listed here.

Depressive symptoms (GDS) (β = −0.39, SE = 0.53, p < 0.001), anxious symptoms (HADS-A) (β = −0.14, SE = 0.48, p = 0.02), physical disability (mRS) (β = −0.40, SE = 1.71, p < 0.001), and level of social support (LSNS) (β = −0.14, SE = 0.17, p = 0.01) were also independent predictors of the HRQoL (SSQoL total score) (**Model 1** and **Table 3**).

### Linking Pathways Between BDES Symptoms and the HRQoL

**Figure 2:**
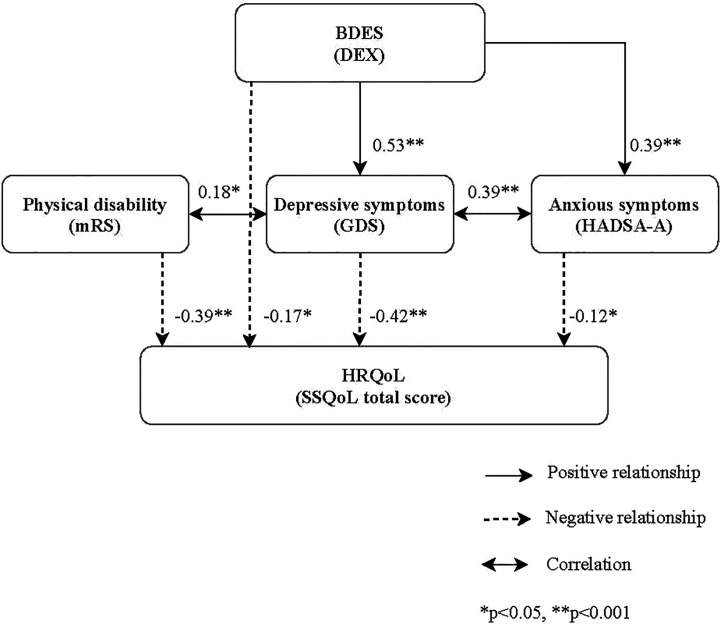
The structural equation model explains the paths from BDES to the HRQoL.

The significant linking pathways from BDES to HRQoL in the SEM model were displayed in **Figure 2**. The CFI (1.00) and RMSEA (<0.001) measures indicated that the final model fitted the data well. In this SEM model, BDES symptoms were not only directly (direct path coefficient of direct effect = −0.17, p < 0.05) but also indirectly (indirect path coefficient = −0.27, p < 0.05) linked with the HRQoL. The depressive symptoms (GDS) (total path coefficient = −0.42, p < 0.001) and anxious symptoms (HADS-A) (total path coefficient = −0.12, p < 0.05) served as the significant mediators. However, BDES symptoms did not contribute to the physical disability (**Figure 2** and **Table S2**).

## Discussion

The present study mainly showed that BDES was associated with worse HRQoL three months after acute ischemic stroke, particularly with the aspects of personality, language, and work/productivity. Moreover, we employed SEM to draw the paths linking BDES to the HRQoL. We observed that BDES was linked with the HRQoL mainly in an indirect manner mediated by emotional disorders rather than physical disability. Our study mirrored and extended findings in stroke population from prior studies in which the association between BDES and the HRQoL has been established in other populations.

Our finding was consistent with previous study results that showed that BDES was associated with lower quality of life in patients with schizophrenia (7), that BDES accounted for inadequate self-efficacy in patients with TBI (21), and that BDES impaired the abilities of daily life in patients with PD (22) and decreased duration of survival in patients with ALS (6). Our prior observation that stroke patients with more severe BDES accompanied with multiple adverse outcomes such as emotional disorders, functional dependence, and poorer cognitive functioning lent further support to the present finding (4). Several lines of evidences may interpret the impacts of BDES on the HRQoL. First, the most common manifestation of BDES was anosognosia (the lack of awareness of cognitive dysfunction) (2), which possibly precluded the patients and caregivers from seeking help and reduced efficacy of life-prolonging therapies likely to result in worse survival conditions among patients with ALS (6). Hypoactivity-apathy, another core feature of BDES (2), correlated with impaired mental quality of life among stroke survivors (23). Second, BDES indicated the impairments in higher order functions such as motivated behavior, inhibition of inappropriate behavior, decision-making, and goal-directing behavior. These psychological processes are the cornerstones of the individuals’ social values and strategies coping with complicated environmental and social situations during work (24). Moreover, those fundamental skills for coping with daily life such as language, prospective remembering, and emotional empathy were suggestive impaired in the context of BDES. In particular, our previous study showed that stroke patients with BDES demonstrated worse verbal fluency and attentional abilities (4); the association between dysexecutive function and comprehension of the complex passive and long sentences were found in patients with PD (22). The stereotyped and perseverative behaviors were associated with impairments of theory of mind and facial emotion recognition suggesting an inability to understand others’ perspectives, beliefs, and emotional feelings (25), leading to maladaptation to daily work and life. Taken together, these evidences also supported the notion that BDES particularly introduced detrimental effects on the domains of language, personality, and work/productivity.

Interestingly, the present study first documented that depressive and anxious symptoms, compared with physical disability, more closely jointed with BDES to be associated with impairment of the HRQoL (4, 6). First, it has been widely observed that depressive and anxious symptoms were robust predictors of impaired quality of life after stroke (26). Meanwhile, patients with BDES were at higher risk of having depression and anxiety among stroke survivors (4), and older adults with late life depression with dysexecutive behavior measured by behavioral rating scales showed greater severity of depression and disability (5). It was plausible that the overlapping damaged brain structures responsible for executive function and emotional regulation serve as underlying link between BDES, depression/anxiety, and the HRQoL. Lesion studies demonstrated that disruption to frontal subcortical circuit was the pivotal cause of BDES (27). Of note, two cross-sectional studies showed that decreased volume of the DLPFC was demonstrated to be related with BDES in patients with schizophrenia or dementia (28, 29). Meanwhile, a large-scale connectomic study has recently confirmed that a brain circuit with DLPFC as the critical node encompassed lesion locations associated with poststroke depression (30). Further, several high-quality studies demonstrated that cognitive behavioral therapy not only effectively alleviate BDES (aggression, impulsivity, and disinhibition) but also anxiety after acquired brain injury (31), giving clue to a common psychological mechanism between BDES and anxiety and further implying the urge to develop strategies targeting both to improve the HRQoL after stroke.

However, in contrast with previous observations (4, 5), we failed to draw a direct relationship between BDES and physical disability. Unlike our previous study (4), the present one owned a smaller study sample. The association between BDES and disability may become insignificant once adjusting with the stronger predictors like depression/anxiety in the SEM. Moreover, the patients with less severe stroke and BDES selected in this study may lead to underestimation of the association between BDES and disability. Nevertheless, this result further supported our main finding that BDES contributed to mainly the mental HRQoL including dimensions of work/productivity and personality instead of the functional dimensions like mobility.

The direct effects were also found in the relationship between BDES and the HRQoL, which agreed with previous finding that apathy, one common feature of BDES, but not depression was a predictor of semantic and phonemic fluency task performance in patients with stroke and transient ischemic attack (32). Further, an imaging study using DTI to tract the white matter fibers in patients with small vessel diseases demonstrated that apathy rather than depression was associated with disruption of white matter integrity in anterior cingulum, fornix and uncinate fasciculus, and that the association between depression and white matter damage disappeared after adjusting apathy, suggesting differential network basis of apathy and depression although both were related to worse quality of life (33). These observations showed that there may be direct paths linking BDES to the HRQoL without the accompany of depression/anxiety.

The findings of the present study should be interpreted cautiously in the context of the limitations and strengths. The major limitation result from the selection bias. A large proportion of stroke patients were excluded due to inabilities to complete the assessments, or refusal to participant in the study, leading to a predominance of patients with mild to moderate stroke. Particularly, most stroke patients were excluded due to lack of MRI scans, which may result from the fact that more severe strokes are usually confirmed by clinical history and computerized tomography, whereas the diagnosis of milder strokes requires confirmation by MRI. Thus, this selection bias collectively lead to less severe BDES in this sample. The findings thus may not be applicable to patients with more severe stroke. The relatively mild severity of BDES could cause under-estimation of strength of associations between BDES and the domains of HRQoL. Second, DEX is a screening questionnaire to track the behavioral problems associated with dysexecutive functioning; therefore, it may not be effective to diagnose the BDES. In addition, GDS is an acceptable tool to measure depressive symptoms after stroke in elderly (17). We should always bear in mind that neither DEX nor GDS could replace a comprehensive clinical assessment. Thus, the implication of the current findings could not extend to clinical BDES or depression. Third, the cognitive executive function was not examined due to our limited human resources. This study could not address whether the cognitive executive function mediated the association between BDES and the HRQoL. Instead, we adjusted the global cognitive function (MMSE score) in the multivariate models, and found that the cognitive function did not contributed to the HRQoL. Moreover, the cross-sectional design precludes us to draw a conclusion regarding causal relationships. Nevertheless, our study has some strengths such as the inclusion of a well-characterized stroke population, the use of standardized assessment instruments, and the application of SEM to delineate the linking pathways.

In conclusion, the present study preliminarily suggested a potential association between the behavioral aspects of dysexecutive functioning and a lower level of the HRQoL, particularly the dimensions of personality, language, and work/productivity evaluated at three months after acute ischemic stroke. Although the BDES symptoms directly impact the HRQoL, they contributed to worse HRQoL mainly through their effects on emotional disorders. These results implicate that stroke patients with both BDES and depressive/anxious symptoms may be at a higher risk of having lower HRQoL. Further studies are warranted to validate these findings.

## Data Availability Statement

The raw data supporting the conclusions of this article will be made available by the authors, without undue reservation.

## Ethics Statement

The studies involving human participants were reviewed and approved by Joint Chinese University of Hong Kong-New Territories East Cluster Clinical Research Ethics Committee. The patients/participants provided their written informed consent to participate in this study.

## Author Contributions

YYL and W-KT designed the study. YYL, LW, and YC collected and analyzed data. YYL drafted the manuscript. LW, YY, VM, WC, and JK revised the manuscript. All authors contributed to the article and approved the submitted version.

## Funding

This study was supported by the National Natural Science Foundation of China (Grant Reference Number: 81371460), by the Fundamental Research Funds for the Central Universities in China (No. 21619349 and No. 21619345) and by Medical Science and Technology Research Foundation of Guangdong Province (No. A2020093).

## Conflict of Interest

The authors declare that the research was conducted in the absence of any commercial or financial relationships that could be construed as a potential conflict of interest.
