# Supplementary material for: Association Between Behavioral Dysexecutive Syndrome and the Health-Related Quality of Life Among Stroke Survivors
Source: Front Psychiatry. 2020 Sep 8;11:563930. doi: 10.3389/fpsyt.2020.563930 (PMC7506061; doi:10.3389/fpsyt.2020.563930)
Supplement: Supplementary file 1 [file Table_1.docx]

**Supplementary Tables**

**Table S1.** Association between demographic, clinical characteristics, neuropsychiatric assessments and SSQoL total score with univariate linear analyses (n = 219).

|  | β^a^ | P |
| --- | --- | --- |
|  |  |  |
| **Demographic characteristics** |  |  |
| Age, years, mean ± SD | -0.01 | 0.86 |
| Female sex, n (%) | -0.09 | 0.16 |
| Education, years, median (IQR) | 0.10 | 0.13 |
| Without spouse, n (%) | -0.02 | 0.74 |
| Current or prior smoker, n (%) | -0.06 | 0.34 |
| **Clinical characteristics on admission** |  |  |
| Hypertension, n (%) | 0.05 | 0.43 |
| Diabetes mellitus, n (%) | 0.004 | 0.95 |
| Hyperlipidemia, n (%) | 0.10 | 0.14 |
| Previous stroke, n (%) | -0.12 | 0.09 |
| Transient ischemic attack, n (%) | -0.01 | 0.88 |
| Ischemic heart diseases, n (%) | 0.01 | 0.85 |
| Atrial fibrillation, n (%) | -0.02 | 0.76 |
| NIHSS, median (IQR) | 0.03 | 0.68 |
| Volume of acute infarcts, mean ± SD, mL | -0.13 | 0.07 |
| **Assessments at 3-month after stroke** |  |  |
| BDEX symptoms (DEX), mean ± SD | -0.47 | <0.001 |
| Depressive symptoms (GDS), median (IQR) | -0.60 | <0.001 |
| Anxiety symptoms (HADS-A), median (IQR) | -0.42 | <0.001 |
| Physical disability (mRS), mean ± SD | -0.49 | <0.001 |
| Social support (LSNS), mean ± SD | 0.14 | 0.04 |
| Cognitive function (MMSE), mean ± SD | 0.20 | 0.003 |

BDEX, Behavioral dysexecutive syndrome; DEX, Dysexecutive Scale; GDS, Geriatric Depression Scale; HADSA-A, Hospital Anxiety Depression Scale (Anxiety subscale); IQR, interquartile range; LSNS, Lubben Social Network Scale; MMSE, Mini–Mental State Examination; mRS, modified Rankin Scale; NIHSS, National Institutes of Health Stroke Scale; SD, standard deviation.

^a^ β indicates the standardized regression coefficients.

**Table S2.** The standardized direct and indirect, and total effects of the predictors contributing to the HRQoL three months after stroke (n = 219).

|  | HRQoL (SSQoL total score) | | | |
| --- | --- | --- | --- | --- |
| Predictors | | Total effect | Direct effect | Indirect effect |
| BDES (DEX) | | -0.44 (100%) | -0.17 (38.6%) | -0.27 (61.4%) |
| Depressive symptoms (GDS) | | -0.42 (100%) | -0.42 (100%) | 0 |
| Anxious symptoms (HADSA-A) | | -0.12 (100%) | -0.12 (100%) | 0 |
| Physical disability (mRS) | | -0.39 (100%) | -0.39 (100%) | 0 |

BDEX, Behavioral dysexecutive syndrome; DEX, Dysexecutive Scale; GDS, Geriatric Depression Scale; HADSA-A, Hospital Anxiety Depression Scale (Anxiety subscale); mRS, modified Rankin Scale.

(CFI=1, RMSE<0.001)
